# DeepDRA: Drug repurposing using multi-omics data integration with autoencoders

**DOI:** 10.1371/journal.pone.0307649

**Published:** 2024-07-26

**Authors:** Taha Mohammadzadeh-Vardin, Amin Ghareyazi, Ali Gharizadeh, Karim Abbasi, Hamid R. Rabiee

**Affiliations:** 1 Department of Computer Engineering, Bioinformatics and Computational Biology Lab, Sharif University of Technology, Tehran, Iran; 2 Faculty of Mathematics and Computer Science, Kharazmi University, Tehran, Iran; The University of Texas, MD Anderson Cancer Center, UNITED STATES OF AMERICA

## Abstract

Cancer treatment has become one of the biggest challenges in the world today. Different treatments are used against cancer; drug-based treatments have shown better results. On the other hand, designing new drugs for cancer is costly and time-consuming. Some computational methods, such as machine learning and deep learning, have been suggested to solve these challenges using drug repurposing. Despite the promise of classical machine-learning methods in repurposing cancer drugs and predicting responses, deep-learning methods performed better. This study aims to develop a deep-learning model that predicts cancer drug response based on multi-omics data, drug descriptors, and drug fingerprints and facilitates the repurposing of drugs based on those responses. To reduce multi-omics data’s dimensionality, we use autoencoders. As a multi-task learning model, autoencoders are connected to MLPs. We extensively tested our model using three primary datasets: GDSC, CTRP, and CCLE to determine its efficacy. In multiple experiments, our model consistently outperforms existing state-of-the-art methods. Compared to state-of-the-art models, our model achieves an impressive AUPRC of 0.99. Furthermore, in a cross-dataset evaluation, where the model is trained on GDSC and tested on CCLE, it surpasses the performance of three previous works, achieving an AUPRC of 0.72. In conclusion, we presented a deep learning model that outperforms the current state-of-the-art regarding generalization. Using this model, we could assess drug responses and explore drug repurposing, leading to the discovery of novel cancer drugs. Our study highlights the potential for advanced deep learning to advance cancer therapeutic precision.

## Introduction

Cancer remains one of the most challenging diseases in modern medicine, with treatments ranging from surgery to chemotherapy and targeted drug therapy. A pharmacological intervention that targets cancer presents fewer side effects than more invasive therapies, but it is also costly, time-consuming, and complex to develop [1–3]. As a result, drug repurposing has emerged as a compelling alternative to traditional drug development [4].

Many clinical methodologies contributed to the advent of drug repurposing. Computational techniques have rapidly eclipsed these traditional approaches, offering new insight by decoding complex relationships between drug data and cancer multi-omics, such as transcriptomics, proteomics, genomics, and epigenomics [5–9]. Early experiments with classical machine learning in drug response prediction have yielded promising results [10–20]. With the ascent of deep learning, its use in cancer drug response prediction has surged, paralleling trends in computational biology [21]. It has been shown that deep learning models can be more accurate than classical models when it comes to predicting drug responses [22–25]. Deep learning’s ability to discern intricate patterns in large datasets makes it one of the most powerful tools for drug response prediction [16,26–29]. Furthermore, deep learning has made it possible to investigate both individual drug response parameters within cancer contexts [29,30] as well as the effect of drug combinations within cancer contexts [4–6]. Furthermore, drug rankings have emerged as one of the key areas of investigation in the cancer drug response analysis [31,32].

Using deep learning techniques to predict drug responses has become increasingly important in recent years due to the capability of handling multi-dimensional feature space and identifying hidden connections between these features. Menden et al. [18] pioneered the use of a feed-forward neural network with a single hidden layer to predict IC50 values. Sharifi-Noghabi et al. [33] proposed a multi-omics late integration approach with triplet loss and a multi-omics data integration approach for enhanced predictions. In recent years, the DeepDRK model has been introduced, and it relies on kernels to extract features from drug and omics data. The present approach, presented by Wang et al. [34], employed a classifier to predict drug response while leveraging the trained model to repurpose the drug. It has also been reported that recommender systems can also be used to predict drug response [35].

Several works have used Convolutional Neural Networks to predict how a patient will respond to a particular medication. In pioneering work in the field, twin neural networks (tCNNs) have been employed to extract features from drug and omics data, representing a novel approach that has been used in the field for many years [36]. There has also been a growing interest in graph-driven drug response prediction (GraphDRP) as a result of the advent of Graph Neural Networks (GNNs) [37]. Liu et al. [38], introduced DeepCDR, a machine learning (GNN) approach to drug response prediction that pioneered the field of predicting drug response. More recent methods have utilized transformers for the task [39,40]. One notable contribution to this field was DeepTTA, which used transformers to extract features and represented a novel approach to drug response prediction [39]. There is another transformer-based method called iBT-Net, which uses transformers to create embeddings of omics data, as well as a simple multilayer perceptron (MLP) to obtain embeddings of drug data to generate a classifier that predicts drug response and uses these embeddings in a classification model [40].

Although these models have made significant progress, they often fail to generalize to different cancer datasets. When trained on specific datasets, such models perform poorly on external datasets. As models struggle to generalize outside specific datasets, the conventional development of predictive algorithms within a single study has frequently led to inflated estimates of prediction performance [29]. Further, a limited variety of data types has constrained the representation of cancer and drugs, highlighting the urgent need for multi-omic integration to enhance representation quality.

As a result, we propose a simpler, autoencoder-based model for generating a feature set for cell lines and drug embeddings using diverse datasets. Based on these embeddings, a classifier can then predict drug responses. In addition to improving generalization, the simplicity of this model enables it to integrate diverse data sources and minimize data noise, thereby enhancing predictions. The following advantages are associated with this approach:

- Comparing autoencoders with more intricate deep-learning models, autoencoders have shown superior generalization abilities.
- By integrating data types, the model was able to produce better results than when using a single omic dataset.
- Using autoencoders for dimensionality reduction improved the model’s performance.
- The model correctly predicted Drug-cell line pairs even though they were not included in the dataset and were unrecognized by the model.

This paper has three sections: the first delineates the datasets employed, the second continues with the problem formulation and explains the proposed solution, the next presents and analyzes experimental results and discusses their correlation with clinical research in detail, and the final section thoroughly discusses these findings.

## Results

We conducted seven experiments to evaluate our model’s performance and generalization capabilities. These experiments covered various scenarios and datasets to assess the model’s robustness thoroughly. In the first experiment, we conducted an ablation study by removing the autoencoder parts of the model to examine their contributions to the final results. The results showed that these autoencoders played a crucial role in the model’s final output.

In the second experiment, we tested the model’s outcome in single omic data with multi-omic integration in generalization, focusing on the data integration hypothesis. Following this ablation study, we started to compare our model with previous methods.

Our third experiment employed a mixed dataset methodology, combining GDSC and CTRP datasets. We used these data as training and testing data, conducted 5-fold cross-validation, and compared our results with DeepDRK. The encouraging results indicated that our model is viable.

In the fourth experiment, we trained the model on a combination of datasets from GDSC and CTRP and tested it on the CCLE dataset to see if it could apply its learned patterns to external datasets. We compared the results of this experiment to those of DeepDRK. We also benchmarked our model against other works in the field, such as DeepTTA [39] and iBT-Net [40], to assess its performance.

We conducted additional experiments to understand the model’s abilities better. In the fifth experiment, we used 5-fold cross-validation to measure the model’s internal consistency as we trained and tested it on the GDSC dataset. In the sixth and seventh experiments, we trained on the GDSC dataset and tested it on the CCLE and CTRP datasets to evaluate its adaptability and efficacy across different datasets.

Our experimental analysis confirmed that our model is resilient and accurate in predicting drug effects. It has the potential to make significant contributions to precision medicine and therapeutic research. The insights from our comparative studies further emphasize the model’s competitive stance in drug response modeling.

### Ablation study

An experiment was conducted to determine the contribution of autoencoder components to the overall effectiveness of a model for drug-cell line interaction classification. Initially, the model was evaluated without autoencoders, using only direct embedding data. As measured by the Area Under the Precision-Recall Curve (AUPRC), the result was a modest 0.74, indicating the need for improvement.

Subsequent trials incorporated the Drug Autoencoder, which led to a slight improvement in performance, with the AUPRC increasing to 0.76. This improvement demonstrated the positive impact of the Drug Autoencoder on the model’s performance.

Then, the Cell Line Autoencoder was evaluated, and it produced a remarkable increase in performance, resulting in an AUPRC of 0.91. This finding highlighted the pivotal role of the Cell Line Autoencoder in refining a feature space from high-dimensional cell line embeddings, which is essential to the model’s architecture.

Finally, the model was tested using both the Cell Line and Drug Autoencoders, and the results showed superior performance, indicating the synergistic effect of using both autoencoders. The results are presented in Table 1.

**Table 1:** Results of the testing model on CTRP+GDSC data set compared to DeepDRK on mixed set test using 5-fold cross-validation.

Model	Accuracy	Precision	Recall	F1 Score	AUC	AUPRC
**DeepDRA(WithoutAE)**	0.49	0.99	0.49	0.65	0.50	0.74
**DeepDRA(DrugAE)**	0.49	0.99	0.49	0.65	0.50	0.76
**DeepDRA(CellAE)**	0.90	0.91	0.88	0.90	0.96	0.96
**DeepDRA**	**0.93**	**0.93**	**0.93**	**0.93**	**0. 97**	**0.97**

Further, we sought to quantify the impact of data integration by comparing the model’s performance on single omics data versus multi-omics data. The comparative analysis revealed that models utilizing multi-omics data outperformed those based on single omics data in mixed-set testing scenarios, as shown in Table 2.

**Table 2:** Results of training model on GDSC and CTRP and test on CCLE.

Experiment	Accuracy	Precision	Recall	F1 Score	AUC	AUPRC
Single mutation	0.803	0.783	**0.857**	0.812	0.890	0.873
Single expression	0.807	0.887	0.806	0.836	0.900	0.876
Single CN	0.801	0.824	0.826	0.820	0.871	0.852
Multi-omic Fingerprints	0.796	0.815	0.827	0.814	0.891	0.875
Multi-omic Descriptors	0.702	0.673	0.772	0.710	0.815	0.824
**Multi-omics**	**0.823**	**0.905**	0.812	**0.850**	**0.909**	**0.896**

### Method comparison

**Fig 1:**
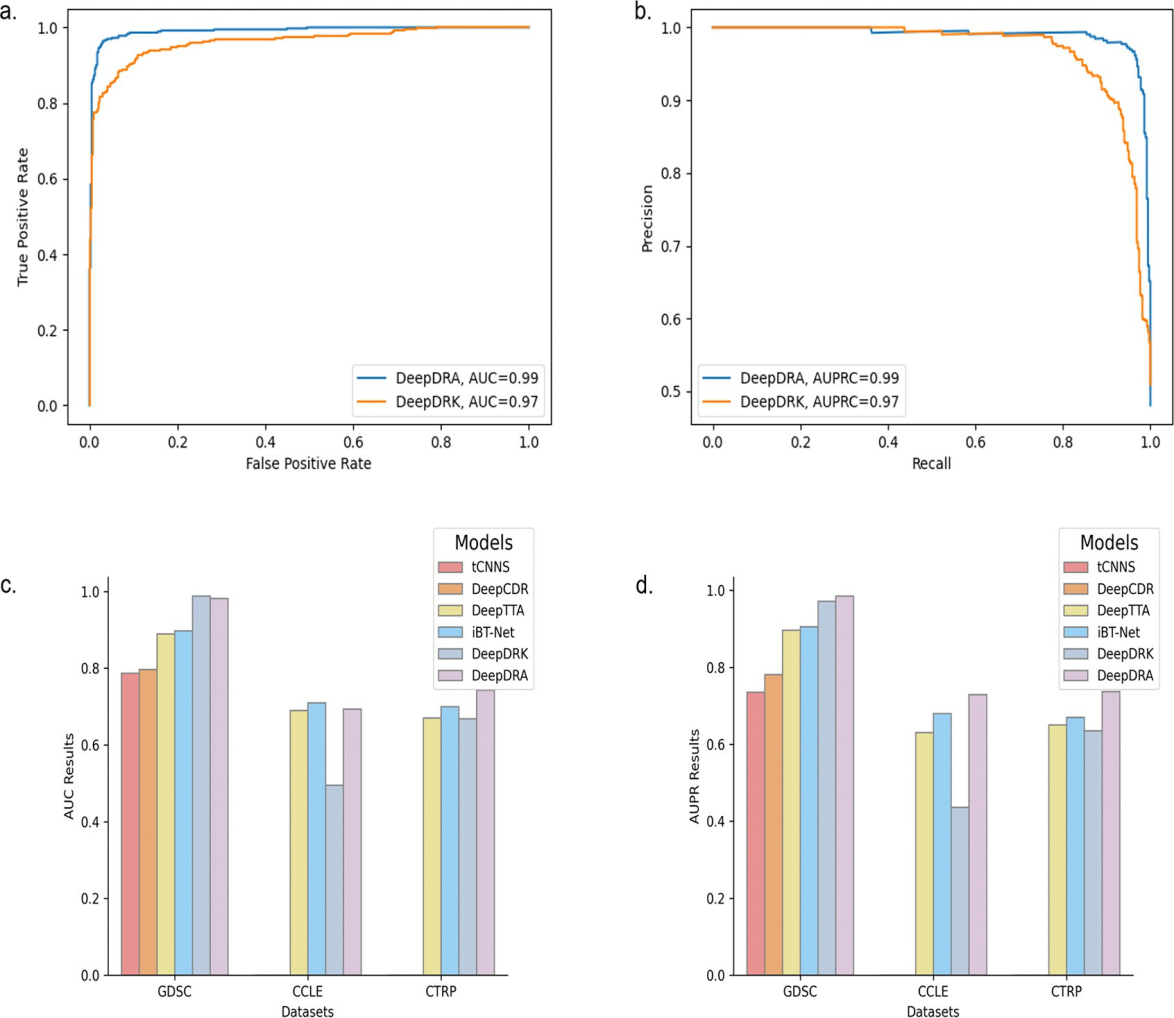
(a) DeepDRA showed improvements in AUC results compared to the DeepDRK model in the mixed-set experiment on the GDSC+CTRP dataset. (b) AUPRC results were reported using the same test set as in (a). (c) DeepDRA and previous models were compared using.

During the initial evaluation, our model was trained on a combined dataset that included expression (exp), mutation (mut), and copy number variation (CNV) data, as well as drug descriptors and drug fingerprints. This dataset helped establish a performance baseline and served as the platform for our model’s first testing phase. Our model showed outstanding performance in this primary assessment with 5-fold cross-validation, achieving an AUPRC of 0.99 (Table 3). We conducted a comparative analysis against the DeepDRK model using an identical dataset to provide context for these results. The DeepDRK model achieved an AUPRC of 0.97, indicating that our proposed model has better results robustness (Fig 1).

**Table 3:** The experiment’s results on the CTRP+GDSC data set were compared to those of the deepDRK on the mixed set test using 5-fold cross-validation.

Model	Accuracy	Precision	Recall	F1 Score	AUC	AUPRC
DeepDRK	0. 832	0. 826	0. 873	0. 815	0. 971	0. 973
**DeepDRA**	**0.953**	**0.945**	**0.96**	**0.952**	**0. 992**	**0.992**

We conducted additional evaluations to determine the capacity of our model to generalize in different scenarios. We performed a cross-dataset analysis where we trained our model on a combined dataset of CTRP and GDSC and then tested it on the CCLE dataset. Our model demonstrated robust generalization capabilities, as evidenced by an AUPRC of 0.87 88 (Table 4). To compare the results, we also subjected the DeepDRK model to the same testing regimen, and it returned an AUPRC of 0.85, which was significantly lower than the performance of our model (Fig 1).

**Table 4:** Results of the training on CTRP+GDSC data set compared to DeepDRK on CCLE as a test.

Model	Accuracy	Precision	Recall	F1 Score	AUC	AUPRC
DeepDRK	0.733	0.970	0.688	0.803	0.840	0.851
**DeepDRA**	**0.804**	**0.832**	**0.823**	**0.823**	**0.883**	**0.887**

These comparative experiments validate the strength and robustness of our model and also highlight its superior ability to generalize across diverse datasets. This attribute is crucial for precision medicine applications.

The model’s performance was rigorously evaluated under various other experimental conditions in these investigations. Our third experiment involved training the model on the GDSC dataset and testing it on the same dataset using 5-fold cross-validation. The model’s proficiency was reflected in an AUPRC of 0.98 (Table 5), a metric that attests to its outstanding predictive accuracy within the GDSC. This performance signifies a notable enhancement over the results achieved in the prior studies.

**Table 5:** Results of training model on GDSC and test on the same dataset using expression and drug data.

Model	AUC	AUPRC
tCNNS	0.787	0.735
DeepCDR	0.797	0.781
DeepTTA	0.889	0.896
iBT-Net	0.897	0.905
DeepDRK	0.988	0.972
**DeepDRA**	0.982	0.985

The model trained on GDSC data was tested against the CCLE dataset to evaluate cross-dataset generalization. Our model results in an AUPRC of 0.73 (Table 6). This outcome affirms the model’s adeptness in extrapolating learned patterns to an external dataset, an advancement that surpasses the generalization capabilities showcased in the preceding research efforts.

**Table 6:** Results of training model on GDSC and test on CCLE using expression and drug data.

Model	AUC	AUPRC
DeepTTA	0.69	0.63
iBT-Net	0.71	0.68
DeepDRK	0.495	0.436
**DeepDRA**	0.693	0.729

The final experiments involved training the model on the GDSC dataset and testing the CTRP dataset. Within this setting, the model manifested results commensurate with established models, thereby underscoring its steadfast performance across distinct datasets. Such consistent efficacy highlights the model’s adaptability and reaffirms its potential as an invaluable tool for drug response prediction (Table 7).

**Table 7:** Results of training model on GDSC and test on CTRP using gene expression and drug data.

Model	AUC	AUPRC
DeepTTA	0.67	0.65
iBT-Net	0.70	0.67
DeepDRK	0.668	0.635
**DeepDRA**	0.743	0.737

The results of our comprehensive experiments show the model’s capacity to function effectively across diverse analytical scenarios. These accomplishments hold significant promise, potentially paving the way for substantial contributions to precision medicine and therapeutic strategy optimization.

To evaluate the model’s generalization, experiments were performed on patient data. Due to the different domains in cell-line data and patient data, it’s normal to observe weak results when testing on actual patient data. Training and testing the model on patient data is preferable to achieve better results. However, patient omic and response data are scarce, and deep learning models require more data to train properly. Due to these challenges, the model was trained on the CTRP+GDSC dataset and tested on TCGA patient data downloaded from the TCGA portal. The results were then compared to DeepDRK, as shown in Table 8. Although the results are low, our models performed better than DeepDRK on patient data.

**Table 8:** Results of training model on CTRP+GDSC and test on TCGA using gene expression and drug data.

Model	AUC	AUPRC
DeepDRK	0. 506	0. 619
**DeepDRA**	**0.527**	**0. 647**

### Drug repurposing

After training our model with the combined CTRP and GDSC datasets, we designed another experiment on breast cancer for further analysis. Our study aimed to evaluate the effectiveness of the full spectrum of drugs within the dataset against this specific type of cancer. Intriguingly, the model identified several effective drugs not previously cataloged in the dataset as sensitive to or resistant to breast cancer.

During validation, the model was tested on curated subsets of cancer cell lines, focusing primarily on breast cancer. Following model predictions for all possible drug-cell line pairs, we ranked the drugs according to their predicted efficacy scores. Those drugs that achieved scores above 0.5 were considered to have a probable positive effect on the cancer type. The high-ranking drugs in our model are commonly used in cancer treatment, followed by specific drugs that we suggest as potential breast cancer therapies. As shown in the following table (Table 9), these drugs, and the relevant references that support their effectiveness are listed.

**Table 9:** Drugs predicted using DeepDRA.

Drug	Cancer	Cell line	Publications
NVP-TAE684	Breast	HCC1500	[41]
Olaparib	Breast	HCC1500	[42]
dabrafenib	Breast	HCC1500	[43]
Bleomycin	Breast	HCC1500	[44]
COL-3	Breast	HCC1500	[45]
LRRK2-IN-1	Breast	HCC1500	[49]
ML162	Breast	HCC1500	[51]
gefitinib	Breast	HCC1500	[46]
JQ-1	Breast	HCC1500	[50]
tigecycline	Breast	HCC1500	[48]
neratinib	Breast	HCC1500	[47]
BRD-A71883111	Breast	HCC1500	NA
ML050	Breast	HCC1500	NA
Venetoclax	Lung	HCC827	[52]
ascorbate	Lung	HCC827	[53]
Temsirolimus	Lung	HCC827	[54]
importazole	Lung	HCC827	NA
methylstat	Lung	HCC827	[55]
blebbistatin	Lung	HCC827	[56]
Schweinfurthin A	Colon	SW1417	NA
dexamethasone	Colon	SW1417	[57]
sotrastaurin	Colon	SW1417	[58]
nilotinib	Colon	SW1417	NA
tosedostat	Colon	SW1417	[59]
foretinib	sarcoma	SK-UT-1	NA
cytarabine hydrochloride	sarcoma	SK-UT-1	[60]
foretinib	sarcoma	SK-UT-1	[61]
gossypol	sarcoma	SK-UT-1	NA
momelotinib	sarcoma	SK-UT-1	[62]

For example, our model indicated that NVP-TAE684 is sensitive to breast cancer, a finding confirmed by recent studies [41]. Similarly, Olaparib, which lacked binarized response data in our database, is now recognized as a breast cancer therapeutic [42]. Dabrafenib also received a high score in the model, which reflects recent research confirming its efficacy against breast cancer [43]. Bleomycin also received a high score for treatment potential despite being binarized as ’unknown’ in our training data, which is consistent with prior investigations [44]. In the following studies, COL-3 was suggested as a potential treatment, a suggestion that was substantiated by recent findings [45]. Our model listed and recommended the following drugs based on their respective scores: LRRK2-IN-1, ML162, gefitinib, JQ-1, tigecycline, and neratinib [46–51].

Our model also identified BRD-A71883111 and ML050 as potential therapeutic agents in addition to the aforementioned drugs. This presents an opportunity for groundbreaking research since these drugs have not yet been explored in the literature. Without empirical data on these compounds, rigorous clinical testing is required to evaluate their efficacy and safety profiles. The recommendation of these drugs adds a new dimension to the ongoing search for innovative breast cancer treatments. It highlights the importance of integrating computational models into the early stages of drug discovery.

## Materials and methods

This section aims to discuss the formulation of the problem and the implementation and evaluation of the model.

### Dataset

Our data acquisition concentrates on three primary datasets in the current study. The Genomics of Drug Sensitivity in Cancer (GDSC) [63] stands out as a comprehensive repository, encompassing comprehensive multi-omics data related to cancer research. This dataset is notably rich, containing critical information on gene mutations, gene expression, copy number variations (CNV), and methylation profile, each contributing to the complexity of oncological molecular characterization. Despite its lack of multi-omics data, the Cancer Therapeutics Response Portal (CTRP) [64,65] is an invaluable source of drug response data. In contrast, the Cancer Cell Line Encyclopedia (CCLE) [66,67] provides comprehensive multi-omics data, including gene expression, mutation profiles, and copy number variation (CNV) data. We complemented CTRP’s lack of multi-omics data by leveraging CCLE’s data to enhance drug response analysis.

### Preprocessing

During the first step of our data preprocessing, we carefully examined all the missing values in our datasets. Whenever we came across NaN (Not a Number) values, we used a standard imputation strategy to replace them with zeroes. This method works well for our datasets, as it helps to keep any distortion of the underlying data distributions to a minimum. Subsequently, we proceeded to extract relevant features from two comprehensive multi-omics datasets. The critical task here was identifying and extracting intersecting features across these datasets. This helped us train and test our model on different datasets.

### Cell-line data

The GDSC dataset contains a vast amount of information about cancer cell lines. It includes 17,738 unique gene expression features, 23,189 features related to mutations, 14,729 features related to methylation, and 21,879 features related to copy number variations. This information can be used to analyze genomic alterations, epigenetics, and structural genomic variances that may be important in the development and progression of cancer. The CCLE dataset comprises 19,221 gene expression features and 19,534 mutation features. The CNV data has 59,267 features, which provides a comprehensive source for understanding the genomic structural diversity of cancer cell lines. Since the CTRP dataset lacks multi-omics data, the CCLE cell-line data was adopted to integrate vital information with CTRP’s drug response data.

A meticulous curation process is undertaken to optimize the datasets for model training. This process involves aligning the three common modalities across the GDSC and CCLE datasets to refine the dataset and eliminate irrelevant or unnecessary data. The outcome is a set of intersecting features that includes 15,963 gene expression features, 17,671 mutation features, and 23,874 CNV features, culminating in a comprehensive feature set of 57,508 for the combined cell-line dataset. By interlinking data from the GDSC and CCLE, we established a foundation for exploring the complex interplay between genetic, epigenetic, and transcriptional elements. Such a systematic approach is essential for unraveling the nuances of cancer biology and drug sensitivity, propelling the frontiers of precision medicine and therapeutic innovation (Table 10).

**Table 10:** The number of features and modalities of each dataset is in a single dataset and combined into two datasets.

Dataset	Gene Expression	Mutation	Methylation	CNV
CCLE	19221	19534	-	59267
GDSC	17738	23189	14729	24503
**CCLE+GDSC**	**15963**	**17671**	**-**	**23874**

### Drug data

To thoroughly study drug responses, it is essential to have access to comprehensive pharmacological data. To this end, we have compiled drug response information from three different datasets: GDSC, CTRP, and CCLE. This compilation has resulted in creating a repository of drug data, laying a solid foundation for conducting in-depth pharmacological analysis. To ensure consistency and accuracy in our analysis, we obtained the SMILES representations for each compound from the PubChem database [68]. These representations provide a standardized chemical notation, which simplifies subsequent computational tasks.

Using the RDKit cheminformatics software, we extracted various molecular descriptors for each drug in our dataset (Landrum). These descriptors encapsulate a range of chemical properties, from structural attributes to physicochemical factors, that indicate the drug’s biological activity and pharmacodynamics. We also extracted Morgan’s Fingerprint data for these drugs and added it to our model. This extraction process resulted in a dataset consisting of 704 unique drug entities. Each entity is defined by a multi-dimensional feature set that reflects its distinct pharmacological profile. We took great care in curating and extracting comprehensive features from drug data, which are essential to this research. We significantly improved the reliability and accuracy of the drug prediction model.

### Drug response

A crucial step in our preprocessing was the binarization of drug response data. The aim was to standardize various response metrics, such as Area Under the Curve (AUC) and half maximal Inhibitory Concentration (IC50), into binary categories that indicate ’resistant’ or ’sensitive’ responses. This binarization process facilitated a uniform analysis framework that can be used for different types of drug responses encountered in our research. In the CTRP dataset, we consider AUC values lower than 6 to be sensitive, while values higher than 16 are considered resistant. For the GDSC dataset, we define drug response values lower than 0.2 as sensitive and values higher than 0.99 as resistant. Finally, for the CCLE dataset, we used the ActArea measurement for binarization. We consider act area values higher than 2 sensitive and values lower than 0.5 resistant. In the final preprocessing stage, we compiled a comprehensive list of drugs from the primary drug response datasets. For each drug, we systematically retrieved its corresponding Simplified Molecular Input Line Entry System (SMILES) [69] representation.

To train our model, we integrated drug response information from two datasets, GDSC and CTRP. This created a large merged dataset with over 28,000 cell-line drug pairs. This dataset was divided into 10,007 pairs labeled as ’sensitive’ and 18,758 pairs labeled as ’resistant’ (Table 11).

**Table 11:** Categorized sample sizes in each dataset.

Drug Response	Sensitive	Resistant
CTRP	1423	6827
GDSC	10820	13331
CCLE	2956	3792
**CTRP+GDSC**	**10007**	**18758**

Before training our classifier, we carefully processed and classified the drug response data to establish a strong foundation. By converting the continuous drug response measurements into binary values, we were able to develop a robust classifier that could generalize well across various cell lines. This approach aligns with standard practices in the field and offers insights into the mechanisms that govern drug sensitivity and resistance. These insights can be invaluable in advancing precision medicine and developing new therapeutic interventions.

### Problem formulation

Our research utilizes diverse inputs, including multi-omics data from cell lines (such as genomics, transcriptomics, and epigenomics), drug descriptors, and fingerprints. The objective of our model is to predict a sensitivity or resistance score, ranging from 0 to 1, for a given drug-cell-line pair. Specifically, we have a dataset represented as a for i ranging from 1 to N, representing the cell-line data, corresponding to the drug descriptor, and denoting the response value associated with this pair. To make predictions, our approach integrates mutation, gene expression, copy number variation, methylation, drug descriptors, and fingerprints.

As part of our approach, we aimed to improve the model’s performance by ensuring that the features were distributed across the four types of omic data utilized in our study. This illustrates our proposed approach’s structure. During our initial phase, we focused on collecting and preprocessing omics datasets. We converted drug response measurements into binary classes and created a processed dataset that could be fed into our model. Next, we obtained multi-omics data for each cell line and used this information to train our cell-line autoencoder. The autoencoder was responsible for distilling the data into a feature space that captured the essential attributes of the multi-omics information.

**Fig 2:**
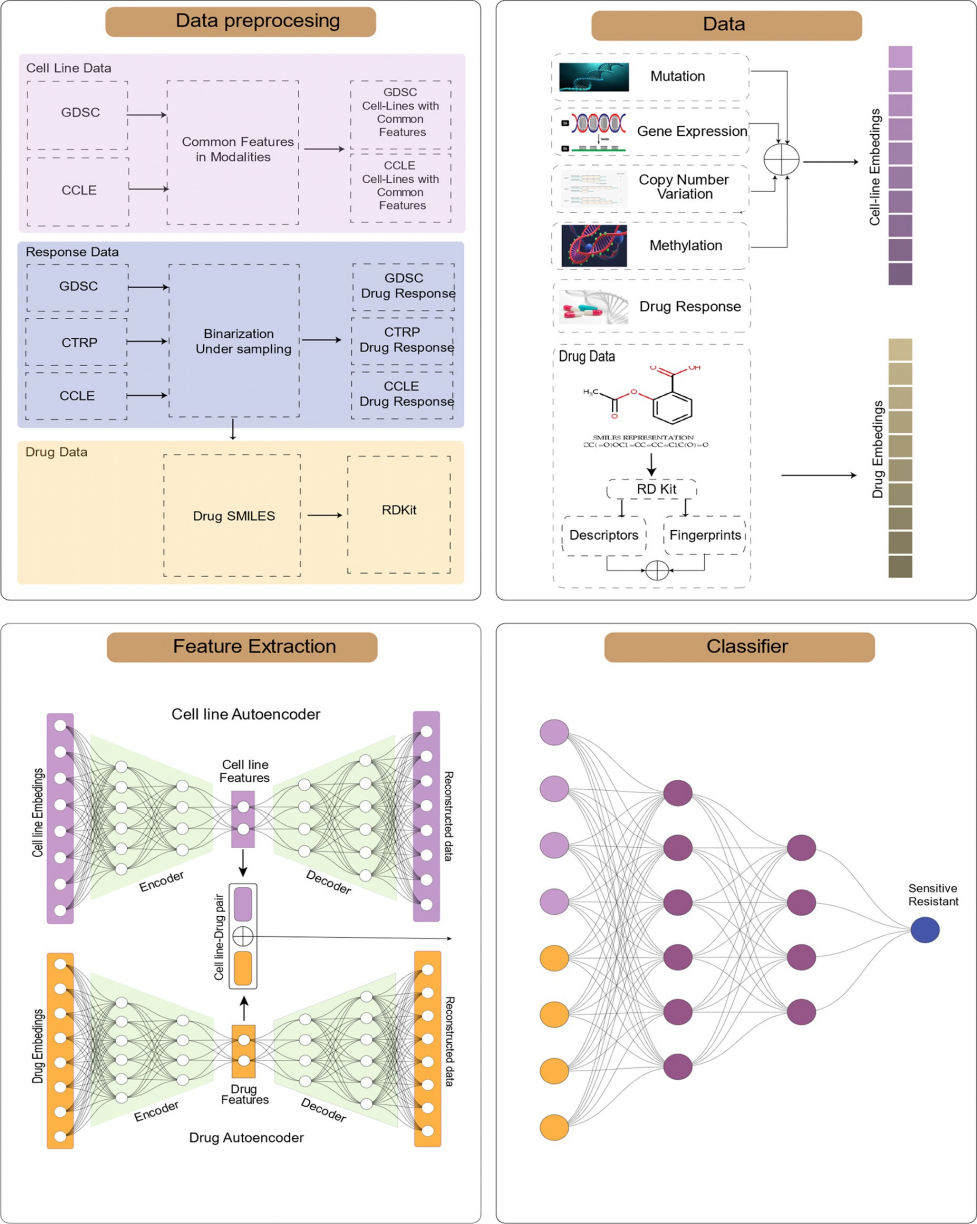
This model involves a multi-step process. Initially, data is collected and preprocessed, cell-line modalities are combined, and the drug embeddings are derived using RDKit. As a final step, autoencoders are utilized to extract features, and then drug response is predicted using a multilayer perceptron (MLP). The figure illustrates these sequential steps.

We have created a system to predict drug responses in cell lines. To do this, we assembled the SMILES for the drugs we are studying and used RDKit to generate a set of molecular descriptors and fingerprints. These drug embeddings were put into our Drug Autoencoder, which created a feature space for each drug entity. We then combined the cell-line data with the drug data to create matched pairs according to drug response pairs. Finally, these pairs were used as input for the final classification model to predict drug responses (Fig 2). We provide further explanation of our approach in the following sections.

### Model implementation and evaluation

In this study, we aimed to improve the generalization capabilities of models within the limited dataset context by using autoencoder architectures. To train the autoencoder and classifier simultaneously, we designed a multi-task model, and its loss function was a composite of the autoencoder and classifier losses. This dual-faceted approach showed the model’s adeptness in handling mixed datasets. Our research objective was to achieve robust generalization, particularly under the constraints of limited data availability. By refining the integration strategies and identifying the most efficient autoencoder architectures, we significantly solved the generalization issue, leading to superior model performance, especially in cross-dataset evaluations. These results have important implications for precision medicine and therapeutic research, as they underscore the potential to enhance our understanding of drug response dynamics.

The autoencoder utilized in this study is designed with a solitary, hidden layer that has 256 units and uses the Rectified Linear Unit (ReLU) activation function. The size of the input data determines the input layer of the autoencoder. The autoencoder employs Mean Squared Error (MSE) as the loss function during training. The model includes the MLP and is trained with a composite loss function that sums the MSE and MLP losses. This multi-task training approach leverages the autoencoder’s feature extraction capabilities and the MLP’s classification strength, creating an effective learning method.

The MLP used in this study consisted of a single hidden layer with 128 units. ReLU and Sigmoid functions were used for the hidden and output layers. The size of the autoencoders’ latent layers determined the size of the input layer. The MLP was trained for 25 epochs using the AdamDelta optimizer with a learning rate of 0.01. The model was trained using a Core i3-10300 CPU and a 3060 Ti GPU. Care was taken to avoid overfitting during the training process by splitting the data into training, validation, and test sets in a ratio of 0.7, 0.1, and 0.2, respectively. Checking these three parts’ accuracy helped avoid overfitting during the training process.

For the loss function, we use MSE loss in autoencoder; the loss function in autoencoder is simple like this:

For the loss function, we use MSE loss in autoencoder; the loss function in autoencoder is simple like this:

$$L_{Autoecoders}=\frac{1}{n}\sum_{1}^{n}{\left({Y_{i}}_{autoencoder}-{{\hat{Y}}_{i}}_{autoencoder}\right)}^{2}$$ (1)

And MLP has Binary Cross Entropy loss:

$$L_{Classifier}=-\frac{1}{n}\sum_{1}^{n}{y_{i}}_{classifier}.log(p({y_{i}}_{classifier}))+(1-{y_{i}}_{classifier}).log(1-p({y_{i}}_{classifier}))$$ (2)

But in end-to-end integration, our loss is like this where Y is for autoencoder and y is the final class:

$$L=L_{Autoencoders}+L_{Classifier}$$ (3)

$$L=\frac{1}{n}\sum_{1}^{n}{\left({Y_{i}}_{autoencoder}-{{\hat{Y}}_{i}}_{autoencoder}\right)}^{2}-\frac{1}{n}\sum_{1}^{n}{y_{i}}_{classifier}.log(p({y_{i}}_{classifier}))+(1-{y_{i}}_{classifier}).log(1-p({y_{i}}_{classifier}))$$ (4)

Hyperparameter optimization was performed through various experiments, and it was determined that an autoencoder with a hidden layer of 256 nodes produced better results than hidden layers with 1024, 512, and 128 nodes. A latent layer with 50 nodes was the best choice among 200, 100, 50, and 25 nodes. For the MLP, a hidden layer consisting of 128 nodes was optimal. Different learning rates were tested, with rates of 0.005, 0.01, and 0.05. It was determined that a learning rate of 0.01 produced the best results. The combination of autoencoder loss and classifier loss with different factors was also tested. Factors a, b, and c were used. The values 2, 1, and 0.5 were tested for each factor, but it was discovered that using a factor of 1 for all losses was the best.


$$L={aL}_{Drug\;autoencoder}+{bL_{Cell-line\;autoencoder}+cL}_{Classifier}$$ (5)

The model code is available on GitHub for model implementation in practical systems and clinical use. First, the model should be trained on the input data. Clinical patient data is preferred over cell-line data for training and achieving better results, but both datasets can be used for training. The model can be trained on different hardware, and using a GPU is not necessary for the training step. After training, the saved model can be used with any patient omics data and all combinations of drugs to find the best drugs for that patient.

## Discussion

We investigated by gathering cell line and response data from three leading cancer databases: GDSC, CTRP, and CCLE. We preprocessed at the initial stage to make the data fit for integration. Our approach involved utilizing four different types of omics data in combination with two categories of drug data, which helped improve prediction accuracy. We ensured that the omics features were consistent across all datasets and eliminated any cases of incomplete data. Moreover, we used the DeepDRK methodology to binarize drug responses, setting thresholds to enable analysis of various datasets and response metrics.

Although there is a current trend in developing complex architectures such as GNNs and Transformers for model development, we prioritized generalizability, especially in cross-dataset validations. For this reason, we designed a streamlined architecture consisting of two main Autoencoders—one for omic data and the other for drug data. We used Mean Squared Error (MSE) loss to reconstruct data in these Autoencoders. The resulting latent representations were then concatenated to create the input for a binary classification classifier. The classifier used binary cross-entropy as its loss function, and the overall loss was calculated by adding the losses of both autoencoders with the loss of the classifier.

Although complex architectures such as GNNs and Transformers are currently trending for model development, we focused on developing a streamlined architecture that prioritizes generalizability, especially in cross-dataset validations. Our architecture consists of two main Autoencoders—one for omics data and the other for drug data—using Mean Squared Error (MSE) loss to reconstruct data. We then concatenated the resulting latent representations to create the input for a binary classification classifier. The classifier used binary cross-entropy as its loss function, and the overall loss was calculated by adding the losses of both autoencoders with the loss of the classifier.

Our research on drug repurposing has led us to compare our latest model with other models in drug response prediction, such as iBT-Net. Our model has consistently outperformed these in various metrics. For example, when trained on 80% of the GDSC dataset and tested on the remaining 20%, our model achieved an AUPRC of 0.98, significantly higher than iBT-Net’s 0.90. Similarly, upon training with GDSC and testing on CCLE, our model achieved an AUPRC of 0.72, compared to iBT-Net’s 0.68, and an AUPRC of 0.73 against CTRP, where iBT-Net scored 0.67.

To validate the clinical relevance of our model, we selected cell lines specific to particular cancers. We tested all plausible drug-cell line combinations not included in our training dataset. Our model’s scoring of these combinations revealed a correlation with established cancer treatments, underscoring the model’s predictive validity. Interestingly, we identified certain drugs that were unique to specific cancers. These drugs have either been recently recognized for their efficacy or are yet to be explored, suggesting potential new avenues for cancer therapy.

In our work, we encountered some limitations that require attention. When we used binarized response data, we experienced some data loss, as the data class was undefined. Additionally, we could not conduct clinical experiments due to incomplete data modalities, which prevented us from testing our model on patient data with all the modalities used in the model.

Based on our findings, we need to focus more on data preparation and integration rather than relying solely on complex models, which may not generalize well. In the future, we plan to improve the Autoencoders’ denoising capability, include additional omics data such as PPRA and RNA-seq, and increase the model’s robustness by training it with datasets that exhibit incomplete modalities. Adding a KEGG pathway or complete survival analysis will be very helpful and should be done in future work.
